# Hybrid Techniques of X-ray Analysis to Predict Knee Osteoarthritis Grades Based on Fusion Features of CNN and Handcrafted

**DOI:** 10.3390/diagnostics13091609

**Published:** 2023-05-02

**Authors:** Ahmed Khalid, Ebrahim Mohammed Senan, Khalil Al-Wagih, Mamoun Mohammad Ali Al-Azzam, Ziad Mohammad Alkhraisha

**Affiliations:** 1 Computer Department, Applied College, Najran University, Najran 66462, Saudi Arabia; 2 Department of Artificial Intelligence, Faculty of Computer Science and Information Technology, Alrazi University, Sana’a, Yemen

**Keywords:** deep learning, knee osteoarthritis, fusion features, handcrafted features, FFNN

## Abstract

Knee osteoarthritis (KOA) is a chronic disease that impedes movement, especially in the elderly, affecting more than 5% of people worldwide. KOA goes through many stages, from the mild grade that can be treated to the severe grade in which the knee must be replaced. Therefore, early diagnosis of KOA is essential to avoid its development to the advanced stages. X-rays are one of the vital techniques for the early detection of knee infections, which requires highly experienced doctors and radiologists to distinguish Kellgren-Lawrence (KL) grading. Thus, artificial intelligence techniques solve the shortcomings of manual diagnosis. This study developed three methodologies for the X-ray analysis of both the Osteoporosis Initiative (OAI) and Rani Channamma University (RCU) datasets for diagnosing KOA and discrimination between KL grades. In all methodologies, the Principal Component Analysis (PCA) algorithm was applied after the CNN models to delete the unimportant and redundant features and keep the essential features. The first methodology for analyzing x-rays and diagnosing the degree of knee inflammation uses the VGG-19 -FFNN and ResNet-101 -FFNN systems. The second methodology of X-ray analysis and diagnosis of KOA grade by Feed Forward Neural Network (FFNN) is based on the combined features of VGG-19 and ResNet-101 before and after PCA. The third methodology for X-ray analysis and diagnosis of KOA grade by FFNN is based on the fusion features of VGG-19 and handcrafted features, and fusion features of ResNet-101 and handcrafted features. For an OAI dataset with fusion features of VGG-19 and handcrafted features, FFNN obtained an AUC of 99.25%, an accuracy of 99.1%, a sensitivity of 98.81%, a specificity of 100%, and a precision of 98.24%. For the RCU dataset with the fusion features of VGG-19 and the handcrafted features, FFNN obtained an AUC of 99.07%, an accuracy of 98.20%, a sensitivity of 98.16%, a specificity of 99.73%, and a precision of 98.08%.

## 1. Introduction

The human body contains many joints, the most important of which is the knee joint. The knee joint connects the thigh with the leg. KOA is one of the most common musculoskeletal system diseases, and it is a chronic disease that leads to disability in the elderly. This disease causes joint pain and progressive knee weakness, which affects more than 5% of people worldwide [1]. There is no effective treatment for KOA, especially when it is of severe grade [2]. Significant factors cause KOA, such as ageing, obesity, and accidental knee injuries [3]. Lack of an early diagnosis of KOA leads to the progression of the disease to a severe grade, in which a complete knee replacement is required. Not all patients with KOA can replace the knee because of its high cost and short life, especially for obese people. Therefore, early diagnosis of KOA is necessary to start treatments that stop KOA from progressing to its dangerous stages and to start drug and behavioral therapies such as weight loss and knee exercises. There are many medical diagnostic imaging techniques. CT scan significantly impacts diagnosing the digestive and respiratory systems, but it has little effect on the bones and revealing the internal spaces. PET imaging is effective in detecting cancer cells at the micro level. Radiography (X-rays) is the gold standard for diagnosing KOA due to its low cost, safety, and availability. Although X-rays are important, they are not sensitive to early detection of changes in OA. Experts use the KL grading method for X-ray diagnosis of KOA to describe the severity and progression of KOA [4]. Joint space width (JSW) is a vital indicator for diagnosing the integrity and severity of the KOA meniscus. In recent years, the International Society’s research on KOA has created an Atlas of Arthritis Research based on the JSW characteristic [5]. Thus, due to the lack of an accurate imaging technique for diagnosing KOA, the diagnosis of X-rays relies on highly experienced physicians to distinguish KL grading for osteoarthritis progression [6]. Thus, the distinction of KL grading is still ambiguous, and doctors’ opinions differ when analyzing X-rays. This ambiguity with manual analysis of X-rays makes early detection of osteoarthritis difficult and thus leads to KOA progressing to the severe stage in which the knee joint must be replaced [7]. Therefore, artificial intelligence techniques solve the shortcomings of manual diagnosis. Artificial intelligence techniques, especially deep learning, aim to reduce uncertainty and reduce human errors [8]. X-rays are a good technique for diagnosing knee osteoarthritis. There are datasets for diagnosing the progression of severe knee osteoarthritis stages, such as the Osteoporosis Initiative and the Rani Channamma University (RCU) used to evaluate the systems in this study. In the classification of X-rays of patients with KOA using deep learning techniques, it has the superior ability to extract complex features and biomarkers that support doctors in providing their diagnosis of the disease condition and accurate prediction of the degree of KOA. To achieve this goal in this paper, many hybrid technologies have been developed that combine various technologies with hybrid features. Because of the symmetry of KL grading and the difficulty of distinguishing between primary grading, features were extracted from more than one deep learning model and combined, removing redundant and unimportant features. Hybrid systems have also been developed to extract features from deep learning models and integrate them with features extracted from traditional methods (handcrafted features).

The purpose of this study is to develop automated systems with the help of artificial intelligence techniques that have the superior ability to help doctors diagnose the progression of the severity of knee osteoporosis initiative and give patients appropriate treatments.

The main contributions to this work are as follows:

- Combining the features of the VGG-19 and ResNet-101 models before and after PCA.
- Combining the features of VGG-19 and ResNet-101 separately with the handcrafted features called fusion features

The remainder of this paper is organized as follows: Section 2 discusses the methods and findings of related work to classify KOA. Section 3 describes the materials and methods used for the X-ray analysis of the two datasets of OAI and RCU of KOA. Section 4 presents the results achieved by the proposed systems for X-ray analysis of the two knee osteoarthritis datasets. Section 5 discusses the performance of the systems and compares their results. Section 6 concludes the paper.

## 2. Related Work

Bayramoglu et al. [9] provided a CNN model for KOA detection from an X-ray image dataset. The BoneFinder tool selected the patella area of interest, and features were extracted using the LBP method to describe the texture of the Region of Interest (ROI). The model reached an AUC of 81.7% and an AP of 48.7%. The model’s performance improved with the ROI, achieving an AUC of 88.9% and an AP of 71.4%. Cheung et al. [10] presented several machine learning algorithms and CNN to analyze knee joint X-rays by analyzing KOA’s KL grade. They provided CNN maps to extract the radiological features that impact the network’s decision. CNN has found better results than machine learning, with an AUC of 99.86%, compared with the best machine learning algorithm, which has an AUC of 41.27%. Tiulpin et al. [11] developed a deep learning model with a Res-Ne architecture for JSW joint space prediction for KOA determination. The model works on segmentation, such as the knee area, to benefit from determining the minimum JSW, and achieved a fragmentation rate of 98.9%. The XGBoost classifier also achieved an AUC of 62.1% by analyzing X-rays to predict KL grade and KOA progression. Javed et al. [12] developed a pre-trained residual network to predict KL grades by analyzing radiographs. A network performance validation was performed with a multicenter dataset. The network achieved an accuracy of 98% and an AUC of 98%. Teo et al. [13] presented the pre-trained InceptionV3 and DenseNet201 models to extract the features of the OAI data set, which is split into five classes according to the severity of the osteoarthritis. Deep learning model features are sent to the SVM classifier for classification. DenseNet201-SVM achieved an accuracy of 71.33%. Tri et al. [14] developed a DCNN for early classification of KOA severity based on analyzing X-rays and extracting features from them. The mesh showed a mean accuracy of 77.24% for each fold of each stage. Yaorong et al. [15] developed a model of clustering algorithm and machine learning to detect knee edges from X-rays to predict the stages of osteoarthritis development. The clustering algorithm works to get data from each X-ray. Complex data was converted to simple for each image and saved to a vector. Finally, machine learning algorithms were applied to analyze the features and predict the severity of OA. Yibo et al. [16] introduced a model consisting of a spatial attention module to improve data extraction from knee X-rays and suppress unnecessary data. Then, the data was merged for all branches of attention units. Mish’s activation function had been set to enable model convergence and improve performance. The model reached an accuracy of 70.23% and F1 scores of 67.55%. Sophal et al. [17] created a model to select ROI from knee X-rays and extract and classify shape features to distinguish between osteoarthritis images and their severity. The ROI was selected by using Otsu’s method from X-rays. Features were reduced by selecting features and feeding them to classifiers to categorize them. The model reached an AUC of 91.7%.

The researchers were able to reach satisfactory results using various methods and materials. The promising accuracy of X-ray image analysis for early detection of KOA remains the goal of every researcher. This study is distinguished from previous studies by the diversity of methods and hybrid materials applied to reach high accuracy. Because of the similarity of KOA in the early stages and the difficulty of determining the intensity of KL grading, this challenge was overcome by extracting the features from more than one deep learning model and combining and then classifying them by FFNN. Moreover, deep learning features were extracted and combined with handcrafted features and then classified by FFNN.

## 3. Materials and Methods

**Figure 1:**
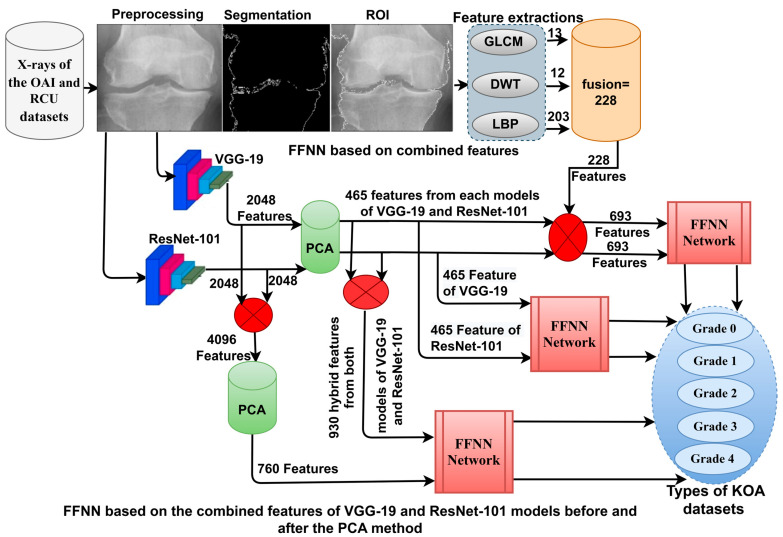
Infrastructure framework for X-ray analysis of the OAI and RCU datasets for discriminating KOA severity grades.

The methodology for X-ray analysis of the OAI and RCU datasets for discriminating KOA severity grades. The following subsections discuss the performance of each method as shown in Figure 1.

The handcrafted features are important for categorizing any image into which class it belongs, but they do not reach a high resolution. Thus, the handcrafted features have limitations in achieving satisfactory accuracy. The advantages of CNN models is their ability to extract subtle and hidden features, and this is what distinguishes them from machine learning. Thus, combining handcrafted features and CNN features will produce representative feature vectors and then achieve promising accuracy.

### 3.1. Description of Two Datasets

**Figure 2:**
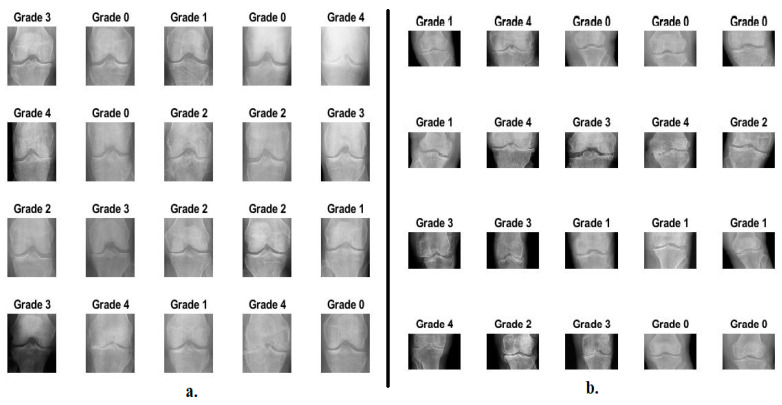
Samples images datasets for all KL grading of osteoarthritis (**a**) from OAI dataset (**b**) from RCU dataset.

Osteoarthritis is a degenerative disease of the articular cartilage of the knee due to the lack of the soft, slippery substance that protects the bones from friction. In this study, the proposed systems were evaluated on the two OAI datasets and the Rani Channamma University (RCU) dataset to analyze X-rays to detect knee arthritis and the severity of KL grading. The first OAI dataset consists of 9786 X-rays divided into five classes according to the severity of the knee joint osteoarthritis according to the KL grading as follows: 3857 X-rays for Grade 0 (Healthy), 1770 X-rays for Grade 1 (Doubtful), 2578 X-rays for Grade 2 (Minimal), 1286 X-rays for Grade 3 (Moderate), and 295 X-rays for Grade 4 (Severe) [18]. The second dataset of the RCU consists of 1650 X-rays divided into five classes according to the severity of the knee joint osteoarthritis according to the KL grading as follows: 514 X-rays for Grade 0 (Healthy), 477 X-rays for Grade 1 (Doubtful), 232 X-rays for Grade 2 (Minimal), 221 X-rays for Grade 3 (Moderate), and 206 X-rays for Grade 4 (Severe) [19]. Table 1 describes the two sets of data and the KOA severity according to KL grading. Figure 2 describes a set of images of the two OAI and RCU datasets for all KL grading of osteoarthritis.

### 3.2. Improving X-ray of Two Datasets of Knee OA

Factors such as the different X-ray machines, surrounding factors, light reflections, movement of the patient’s knee during imaging, and other issues constitute noise in the X-rays, which leads to the deterioration of the performance of artificial intelligence systems. Thus, all these artifacts must be removed and the variance of the knee joint, medial femoral, and osteophytes increased.

In this study, the average filter and Contrast-limited Adaptive Histogram Equalization (CLAHE) method were applied to improve the X-rays of KOA.

First, all X-rays of the OAI and RCU datasets were passed to an average filter to remove noise. The filter selects 16 pixels in each iteration distributed into a target pixel and 15 adjacent pixels. Then, the filter calculates the average of 15 neighboring pixels, removes the value of the target pixel, and replaces it with the average of its neighbors as in Equation (1). The filter continues and processes each pixel in the X-ray [20].

(1) $$f\left(m\right)=\frac{1}{p}\sum_{i=0}^{p-1}s\left(m-i\right)$$

where $f\left(m\right)$ refers to the input, $s\left(m-i\right)$ refers to the old input, and *p* refers to the number of pixels.

**Figure 3:**
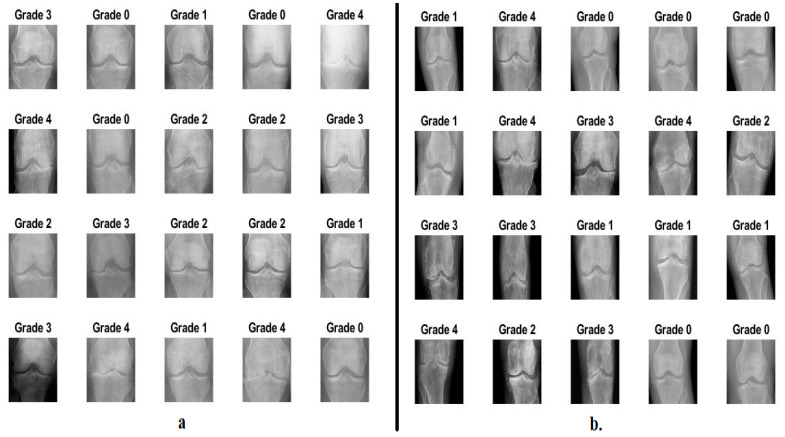
Samples images datasets for all KL grading of osteoarthritis after improvement (**a**) from OAI dataset (**b**) from RCU dataset.

Second, the X-rays were passed after removing the noise to the CLAHE method to increase the visibility of the knee joint and all the bony details adjacent to the knee. The method distributes the bright pixels to the dark areas. Each time the technique compares a target pixel with neighboring pixels, the contrast increases or decreases according to the pixel value of the neighbors [21]. When a pixel’s value is less than its neighbors, its contrast decreases, while its contrast increases when its value is more than its neighbors. The method continues and each pixel is compared to its neighbors to increase or decrease its contrast. Figure 3 shows a set of X-rays of the two OAI and RCU datasets for all KL grading of osteoarthritis after improvement. It should be noted that the images in Figure 2 are the same as in Figure 3 after improvement.

### 3.3. FFNN with CNN Features

This Section 3.3 discusses the techniques and materials applied to analyze the X-rays of the two OAI and RCU datasets to detect the severity grade of osteoarthritis. Training a dataset using CNN models takes a long time, sophisticated computers, and is expensive, and despite this, it may not reach satisfactory accuracy [22]. Therefore, this technique was applied, which consists of two parts: CNN to extract features and FFNN to classify features quickly and accurately.

#### 3.3.1. Extract Deep Features

Artificial intelligence techniques, particularly CNN models, have been inputted in many fields to serve humanity, and the medical side has received a golden share of artificial intelligence techniques. CNN is distinguished by its superior ability in health care, especially in analyzing and processing biomedical images, due to its exceptional ability to extract hidden data [23].

CNN comprises dozens of layers that extract all the data from X-rays of KOA, even hidden, that experts do not see. The essential layers that analyze images to extract their data are convolutional layers, pooling layers, and some auxiliary layers [24]. This study analyzed X-rays of KOA and extracted features using VGG-19 and ResNet-101 models through deep convolutional layers. Convolutional layers are one of the essential layers of CNN, and each layer has a particular task for analyzing and extracting X-ray features. Some layers extract color features, and some focus on extracting the edges of the ROI. Other layers increase the contrast of the crucial areas and there are layers to extract the geometric features, so each layer performs a specific task. In the end, all the features are integrated to produce features representative of each image. Convolutional layers depend on the filter *f*(*t*) size that wraps around the image *x*(*t*) to be processed, as in Equation (2).

(2) $$W\left(t\right)=\left(x*f\right)\left(t\right)=\int x\left(a\right)f\left(t-a\right)da$$

where *W(t)* refers to the output, *f(t)* refers to the size of the filter, and *x(t)* refers to the X-ray inputted.

Convolutional layers produce millions of neurons, which requires computational complexity and long training times. CNN solves this challenge through pooling layers that reduce the number of neurons and weights through two methods, max and average pooling [25]. The max layers select a set of pixels, compare each pixel with the other, select the max value, and put it instead of the selected pixels, as in Equation (3) [25]. The average pooling layers select a group of pixels, calculate its average, and put it instead of the selected pixels, as in Equation (4) [26].

(3) $$z\left(i;j\right)={max}_{m,n=1\ldots .k}f\left[\left(i-1\right)p+m;\left(j-1\right)p+n\right]$$

(4) $$z\left(i;j\right)=\frac{1}{k^{2}}\sum_{m,n=1\ldots .k}f\left[\left(i-1\right)p+m;\left(j-1\right)p+n\right]$$

where *m*, *n* means the location in a matrix, *p* means the stride of the filter, *f* means the filter size, and *k* means the features in vectors.

There are also auxiliary layers after convolutional layers, such as the ReLU layer, which further improves the output by passing positive values and suppressing negative values, as in Equation (5).

(5) $$ReLU\left(x\right)=\max\left(0,x\right)=\left\{\begin{array}{c} x,x\geq 0\\ 0,x<0 \end{array}\right.$$

To avoid overfitting problems, the dropout layer is set to 50%, which passes 50% of the data each time.

The VGG-19 and ResNet-101 models produce features with a size of 9786 × 2048 and 1650 × 2048 for the OAI and RCU datasets, respectively. It is noted that the resulting features are high-dimensional, so these features were passed to the PCA method to delete the redundant and non-significant features and save the essential features with a size of 9786 × 465 and 1650 × 465 for the two datasets of OAI and RCU, respectively.

#### 3.3.2. FFNN Network

FFNN is a highly efficient neural network for solving classification tasks, including medical image processing. Classification tasks are solved through three basic layers. The input layer receives the features sent from the CNN models. The input layer contains 465 input units according to the number of features for each image. The features pass through 15 hidden layers in which complex operations are performed to perform the required tasks. The output layer contains five neurons for each of the two datasets according to the grade of KOA. The data passes in the network from the input layer in the forward direction, and the weights of the neuron in the next layer are calculated according to the value of the previous neuron with its weight. Each time the weight is updated, the minimum square error (MSE) is calculated between the actual $x_{i}$ and expected $y_{i}$ values. The network continues until it reaches the stage of stability, where the weights do not change. Then, the network chooses the weights with the MSE as in Equation (6).

(6) $${MSE=\frac{1}{n}\sum_{i=1}^{n}\left(x_{i}-y_{i}\right)}^{2}$$

where *n* means the number of features, $x_{i}$ means the actual output, and $y_{i}$ means the expected output [27].

**Figure 4:**
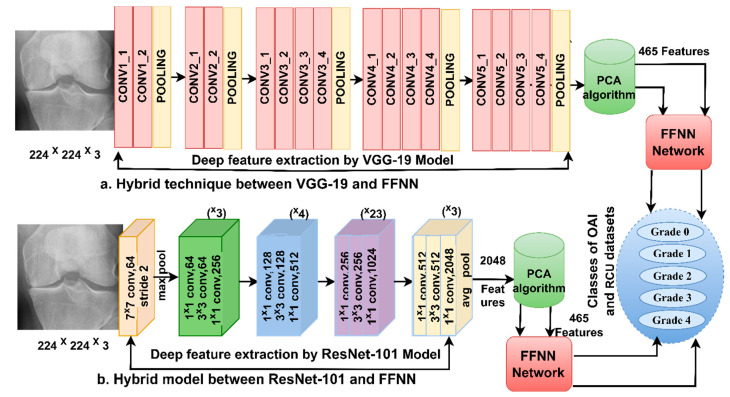
Approaches of the X-ray analysis of the two OAI and RCU datasets for diagnosing osteoarthritis of the knee and discrimination of severity grade using (**a**) VGG-19-FFNN (**b**) ResNet-101-FFNN.

Figure 4 illustrates the X-ray analysis methodology of the two OAI and RCU datasets for diagnosing KOA and discrimination of severity grade of the osteoarthritis by VGG-19-FFNN and ResNet-101-FFNN techniques.

### 3.4. FFNN with Fusion of CNN Features

This section discusses the techniques and materials applied for analyzing X-rays of the OAI and RCU datasets for detecting severity grade of KOA. Training a dataset using CNN models is time-consuming, sophisticated, and requires expensive computers and, despite this, may not reach satisfactory accuracy. So, this technique was applied, consisting of two parts: VGG-19 and ResNet-101 models for feature extraction and merging and FFNN for quick and accurate feature classification [28].

The methodology of this section consists of two systems based on combining the features of VGG-19 and ResNet-101 as follows. The first system extracts features from VGG-19 and ResNet-101 separately, then the features are merged and fed into the PCA to eliminate the repeated and unessential parts and keep the essential features. In the second system, features are extracted from VGG-19 and ResNet-101 separately; then, they are fed into the PCA separately to eliminate those that are redundant and unessential and keep the essential features. Finally, the essential features of the VGG-19 and ResNet-101 models are combined.

**Figure 5:**
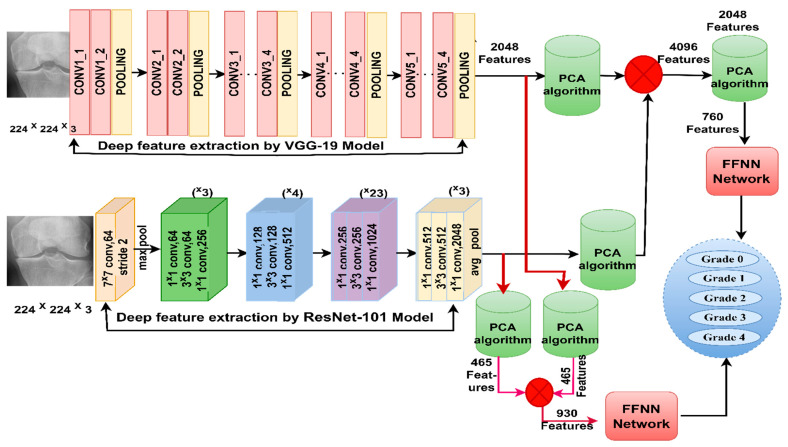
Approaches of the X-ray analysis of the two OAI and RCU datasets for diagnosing osteoarthritis of the knee and discrimination of severity grade using FFNN with fusion features of VGG-19 and ResNet-101.

Figure 5 illustrates the X-ray analysis methodology of the two OAI and RCU datasets for discriminating the severity of osteoarthritis by integrating features of VGG-19 and ResNet-101 before and after PCA.

For the first system, the X-rays of the two OAI and RCU datasets for diagnosis of the severity of grade of KOA are analyzed in several steps as follows.

Firstly, the X-rays were improved, with better appearance of the knee joint through the average filter and CLAHE method. Secondly, the optimized X-rays were fed to the VGG-19 for analysis and extraction of the important and hidden features by convolutional layers, saving them at a size of 9786 × 2048 and 1650 × 2048 for the OAI and RCU datasets of osteoarthritis, respectively.

Thirdly, feeding the improved X-rays to ResNet-101 for analysis and extracting important and hidden features by convolutional layers and saving them at a size of 9786 × 2048 and 1650 × 2048 for the OAI and RCU datasets of osteoarthritis, respectively.

Fourthly, integrating the features of VGG-19 and ResNet-101 and saving them at a size of 9786 × 4096 and 1650 × 4096 for the OAI and RCU datasets of osteoarthritis, respectively.

Fifthly, feeding the merged features of size 9786 × 4096 and 1650 × 4096 to the PCA method to remove redundant and unnecessary features and keep the necessary features of size 9786 × 760 and 1650 × 760 for the OAI and RCU datasets of osteoarthritis, respectively.

Sixthly, feeding essential features with a size of 9786 × 760 into FFNN for training and system performance testing.

Seventhly, feeding the essential features with a size of 1650 × 760 into FFNN for training and system performance testing.

For the second system, the X-rays of the two OAI and RCU datasets for diagnosis of the severity of grade of KOA are analyzed in several steps as follows:

The first three steps of the second system are the same as the first system.

Fourthly, feeding the VGG-19 features into the PCA method to remove redundant and unnecessary features and keep the necessary features at a size of 9786 × 465 and 1650 × 465 for the OAI and RCU datasets of osteoarthritis, respectively.

Fifthly, feeding the ResNet-101 features into a PCA method to remove redundant and unnecessary features and retain the necessary features at a size of 9786 × 465 and 1650 × 465 for the OAI and RCU datasets of osteoarthritis, respectively.

Sixthly, integrating the features of VGG-19 and ResNet-101 and saving them at a size of 9786 × 930 and 930 × 4096 for the OAI and RCU datasets of osteoarthritis, respectively.

Seventhly, feeding essential features with a size of 9786 × 930 and 1650 × 930 for the OAI and RCU datasets of osteoarthritis, respectively, into FFNN for training and system performance testing.

### 3.5. FFNN Network with Hybrid Features of CNN and Handcrafted Features

This section discusses the techniques and materials applied for analyzing X-rays of the OAI and RCU datasets to detect the severity grade of KOA. Training the dataset using CNN models takes a long time, is complicated and costly for computers, and may not reach satisfactory accuracy. So, this technique, which consists of two parts, has been applied: two models of VGG-19 and Resnet-101 to extract the features separately and combine them with the features of GLCM, DWT, and LPB methods.

The methodology of this section consists of two systems that depend on the fusion features extracted in a way that combines CNN features with handcrafted features.

**Figure 6:**
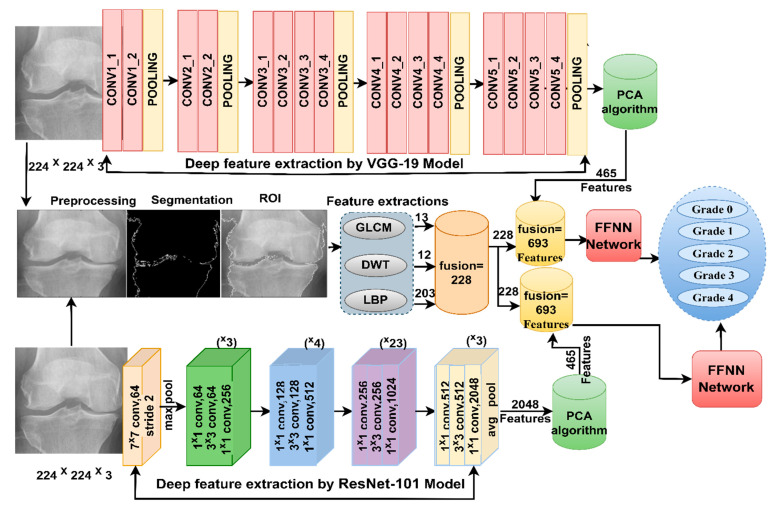
Approaches of the X-ray analysis of the two OAI and RCU datasets for diagnosing osteoarthritis of the knee and discrimination of severity grade using FFNN with fusion features.

Figure 6 shows the methodology of X-ray analysis of the two OAI and RCU datasets for diagnosing and discriminating the severity of osteoarthritis through fusion features of VGG-19 and handcrafted features, in addition to fusion features of ResNet-101 and handcrafted features.

The methodology of this technique for X-ray analysis of the two OAI and RCU datasets to diagnose the severity of osteoarthritis of the knee in several steps is as follows:

First, the X-rays were enhanced, and the contrast of the knee joint was augmented by an average filter and the CLAHE method.

Second, the enhanced knee X-rays were fed to VGG-19 and ResNet-101 separately for analysis and minute and hidden features were extracted by convolutional layers; they were saved at a size of 9786 × 2048 and 1650 × 2048 for the OAI and RCU datasets of KOA, respectively.

Third, feeding features of the VGG-19 and ResNet-101 separately into the PCA method to remove redundant and unnecessary features and keep the necessary features at a size of 9786 × 465 and 1650 × 465 for the OAI and RCU datasets of osteoarthritis, respectively.

Fourth, extracting geometric and texture features through GLCM, DWT, and LBP methods and combining them, which are called handcrafted features [29].

Enhanced X-rays are fed to DWT for extraction and analysis and geometry features. This method has four filters; therefore, the X-rays are divided into four parts for analysis. Each filter serves to analyze one part of the X-ray. The first X-ray part is passed to the low filter to analyze its approximate components and extract three statistical features. The X-rays’ second and third parts are passed to the Low-High and High-Low filters to analyze their detailed components and extract three statistical features from each part [30]. In the fourth part, the X-ray is passed to the high filters to analyze their detailed components and extract three statistical features. Thus, the four filters produced 12 features of size 9786 × 12 and 1650 × 12 for the OAI and RCU datasets of osteoarthritis, respectively.

Enhanced X-rays are fed to the GLCM for analysis and extraction of the texture features of the knee joint. This method converts the X-rays into a grayscale matrix to extract features from the knee area. The method calculates the X-rays’ spatial information based on the neighbors’ distance and angles. The method decides whether an area is rough or smooth depending on the pixel and its neighbors [31]. If the adjacent pixels are close together, the region is smooth. In contrast, the region is rough if the pixels have different values. Thus, GLCM produces 13 features of size 9786 × 13 and 1650 × 13 for the OAI and RCU datasets of osteoarthritis, respectively.

Enhanced X-rays are fed into the LBP to analyze and extract features of the binary surfaces. This method converts the image into a grayscale matrix for feature extraction. The method calculates the spatial information of the X-rays and counts each pixel with its neighbors to start the processing process. In each iteration of processing a target pixel, the method takes 24 adjacent pixels. The method calculates the target pixel and the neighbors according to Equation (7) and replaces the target pixel with the product of the LBP [32]. The method continues until all pixels are completed and replaced according to the LBP method. Thus, the LBP yields 203 features with sizes of 9786 × 203 and 1650 × 203 for the OAI and RCU datasets of osteoarthritis, respectively.

(7) $${LBP}_{R,P}=\sum_{p=0}^{P-1}s\left(g_{p}-g_{c}\right)2^{p}$$

where $g_{c}$ means the center pixel, *R* means the contiguous radius, $g_{p}$ means the contiguous pixels, and *P* means the number of contiguous pixels.

Fifth, the features of the three methods are merged and saved at a size of 9786 × 228 and 1650 × 228 for the OAI and RCU datasets of osteoarthritis, respectively. These are called handcrafted features.

Sixth, the features produced from VGG-19 are combined with the handcrafted features at a size of 9786 × 693 and 1650 × 693 for the OAI and RCU datasets of osteoarthritis, respectively.

Seventh, the features produced from ResNet-101 are combined with the handcrafted features at a size of 9786 × 693 and 1650 × 693 for the OAI and RCU datasets of osteoarthritis, respectively.

Eighth, the essential features with a size of 9786 × 693 and 1650 × 693 for the OAI and RCU datasets of osteoarthritis, respectively, are fed into FFNN for training and system performance testing.

## 4. Experimental Results of the System’s Performance

### 4.1. Split of OAI and RCU Datasets

This study aims to develop hybrid systems with high-efficiency hybrid features to distinguish KOA’s severity grade accurately. The proposed systems were evaluated on X-rays of the OAI and RCU datasets of the knee. The dataset of OAI and RCU contain 9786 and 1650 X-rays, respectively, divided into five grades for the severity of the KOA, as shown in Table 1. In all systems, the two datasets were divided during the systems training phase and validated by 80%, and 20% of the two datasets were allocated for testing the performance of the proposed systems, as shown in Table 2.

### 4.2. Evaluating Systems

The performance of the systems was evaluated through the confusion matrix and the AUC produced by the systems during the X-ray test phase of the two datasets, OAI and RCU, for diagnosing the severity of osteoarthritis. The confusion matrix represents the X-rays during the testing of the two datasets that were correctly analyzed (TN and TP) and the X-rays that were incorrectly analyzed (FN and FP) [33]. The performance of the systems was measured through the evaluation scales mentioned in Equations (8)–(12).

(8) $$AUC=\frac{TPRate}{FPRate}\times 100\%$$

(9) $$Accuracy=\frac{TN+TP}{TN+TP+FN+FP}\times 100\%$$

(10) $$Sensitivity=\frac{TP}{TP+FN}\times 100\%$$

(11) $$Specificity=\frac{TN}{TN+FP}\times 100$$

(12) $$Precision=\frac{TP}{TP+FP}\times 100\%$$

### 4.3. Balancing with Augmentation Data for the Two Datasets

For CNN models to reach good results, they need to be fed with a large dataset to avoid the problem of overfitting. Many biomedical datasets experience a significant shortage of dataset numbers. Moreover, biomedical datasets face the issue of the imbalance of its classes, which makes the accuracy tend to the type of disease (classes) that has more images. Therefore, these challenges are a limitation of CNN models. These limitations were overcome by applying the X-ray data augmentation technique to the OAI and RCU datasets of osteoarthritis. The lack of X-rays for the OAI and RCU datasets was overcome by data augmentation that artificially augments original X-rays. There are many data augmentation method operations, such as rotating, flipping, shifting, and changing the height and width of the X-ray [34]. The problem of an unbalanced dataset was also overcome by increasing the X-rays differently from one class to another.

**Figure 7:**
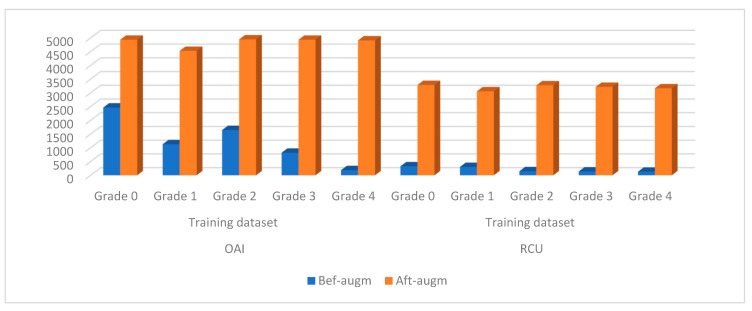
Showing the performance of the data augmentation method to balance the two data sets and overcome the overfitting problem.

Table 3 describes the number of X-rays for the two OAI and RCU datasets for KOA during the training of the dataset before and after the data augmentation was applied. If all classes are increased equally, the dataset remains unbalanced. Therefore, each class will be increased differently from the other class. In this work, it is noted that each category (degree) increased the type of KOA severity by an amount different from the other category to balance the two datasets. Figure 7 shows the distribution of X-rays for the two datasets before and after applying the data augmentation method.

### 4.4. Results of FFNN with CNN Features

This section summarizes the results of the systems for analyzing the X-rays of the OAI and RCU datasets for diagnosing the severity of osteoarthritis before it progresses to the severe stage. The VGG-19-FFNN and ResNet-101-FFNN techniques extract features from CNN models and pass them to the PCA to remove redundant features and maintain important features. The important features are sent to FFNN to split the features of the two datasets to train the systems and test their performance.

Table 4 summarizes the results obtained by the two techniques, VGG-19-FFNN and ResNet-101-FFNN, for X-ray analysis of an OAI dataset and discrimination of a severity grade. The VGG-19-FFNN reached an AUC of 96.88%, an accuracy of 95.8%, sensitivity of 92.99%, specificity of 98.74%, and precision of 92.06%. On the other hand, ResNet-101-FFNN achieved an AUC of 97.76%, an accuracy of 95.10%, sensitivity of 92.31%, specificity of 98.88%, and precision of 91%.

Table 5 summarizes the results obtained by the two techniques, VGG-19-FFNN and ResNet-101-FFNN, for X-ray analysis of an RCU dataset and discrimination of a severity grade. The VGG-19-FFNN reached an AUC of 96.49%, an accuracy of 93.3%, sensitivity of 92.44%, specificity of 98.12%, and precision of 93.4%. On the other hand, ResNet-101-FFNN achieved an AUC of 95.27%, an accuracy of 91.5%, sensitivity of 90.97%, specificity of 97.78%, and precision of 90.96%.

**Figure 8:**
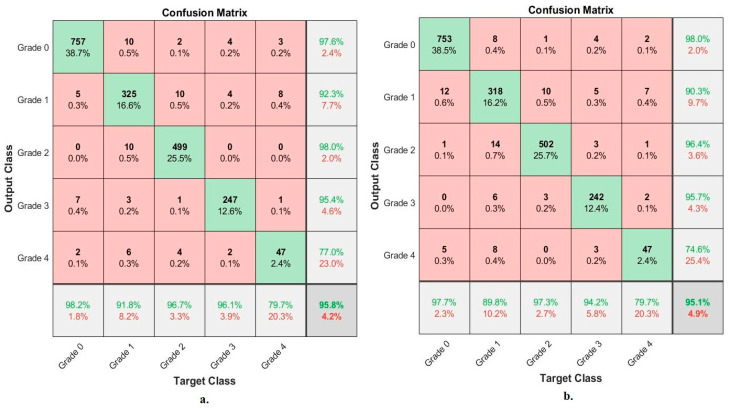
Display of the confusion matrix of the X-ray analysis of an OAI dataset for early diagnosis of severe KOA by FFNN with features of (**a**) VGG-19 (**b**) ResNet-101.

Figure 8 shows the performance of VGG-19-FFNN and ResNet-101-FFNN techniques for X-ray analysis of the OAI dataset and grade-severity discrimination. The VGG-19-FFNN achieved the following accuracies for each grade to distinguish the severity of osteoarthritis: accuracy for Grade 0 of 98.2%, for Grade 1 of 91.8%, for Grade 2 of 96.7%, for Grade 3 of 96.1%, and for Grade 4 of 79.7%. On the other hand, ResNet-101-FFNN achieved the following accuracies for each grade to distinguish the severity of osteoarthritis: for Grade 0 of 97.7%, for Grade 1 of 89.8%, for Grade 2 of 97.3%, for Grade 3 of 94.2%, and for Grade 4 of 79.7%.

**Figure 9:**
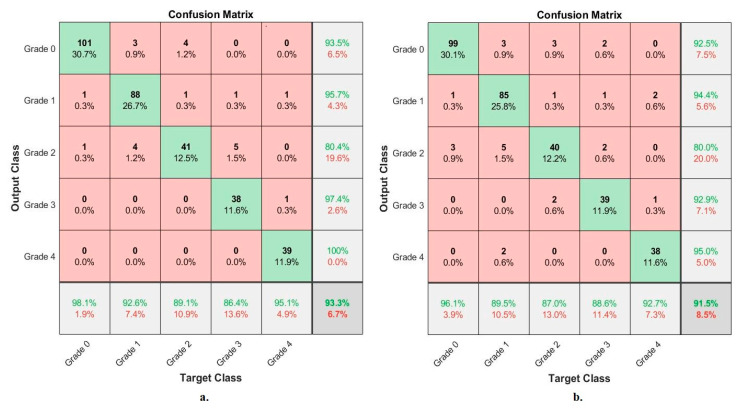
Display of the confusion matrix of the X-ray analysis of an RCU dataset for early diagnosis of severe KOA by FFNN with features of (**a**) VGG-19 (**b**) ResNet-101.

Figure 9 shows the performance of the VGG-19-FFNN and ResNet-101-FFNN techniques for X-ray analysis of the RCU dataset and discrimination of a severity grade. The VGG-19-FFNN achieved the following accuracies for each grade to distinguish the severity of osteoarthritis: for Grade 0 of 98.1%, for Grade 1 of 92.6%, for Grade 2 of 89.1%, for Grade 3 of 86.4%, and for Grade 4 of 95.1%. On the other hand, ResNet-101-FFNN achieved the following accuracies for each grade to distinguish the severity of osteoarthritis: accuracy for Grade 0 of 96.1%, for Grade 1 of 89.5%, for Grade 2 of 87%, for Grade 3 of 88.6%, and for Grade 4 of 92.7%.

### 4.5. Results of FFNN with Fusion of CNN Features

This section summarizes the results of hybrid systems with hybrid features for analyzing X-rays of the OAI and RCU datasets for diagnosing the severity of osteoarthritis before it progresses to the severe stage. Two systems have been developed based on combining the features of VGG-19 and ResNet-101 before and after the PCA method. The idea of this technique is first to extract the features of VGG-19 and ResNet-101 and then integrate the high-dimensional features. Then, the high dimensions are reduced by PCA. Secondly, the technique extracts the features of VGG-19 and ResNet-101 and then reduces their high dimensions separately. Then, the low-dimensional features are incorporated. The important feature is sent to FFNN to split the features of the two datasets to train the systems and test their performance.

Table 6 summarizes the results obtained through FFNN based on the combined features of VGG-19 and ResNet-101 of the OAI dataset and severity grade discrimination. With hybrid features of VGG-19-ResNet-101-PCA, FFNN reached an AUC of 97.49%, an accuracy of 97.1%, a sensitivity of 96.21%, a specificity of 99.48%, and precision of 94.98%. Whereas, with hybrid features of VGG-19-PCA with ResNet-101-PCA, FFNN achieved an AUC of 97.66%, accuracy of 98%, sensitivity of 97.32%, specificity of 99.46%, and precision of 97.12%.

Table 7 summarizes the results obtained through FFNN based on the combined features of VGG-19 and ResNet-101 of the RCU dataset and severity grade discrimination. With hybrid features of VGG-19-ResNet-101-PCA, FFNN reached an AUC of 96.29%, an accuracy of 95.7%, a sensitivity of 95.13%, a specificity of 98.86%, and precision of 95.86%. Whereas, with hybrid features of VGG-19-PCA with ResNet-101-PCA, FFNN achieved an AUC of 96.96%, accuracy of 95.7%, sensitivity of 95.2%, specificity of 98.55%, and precision of 95.04%.

**Figure 10:**
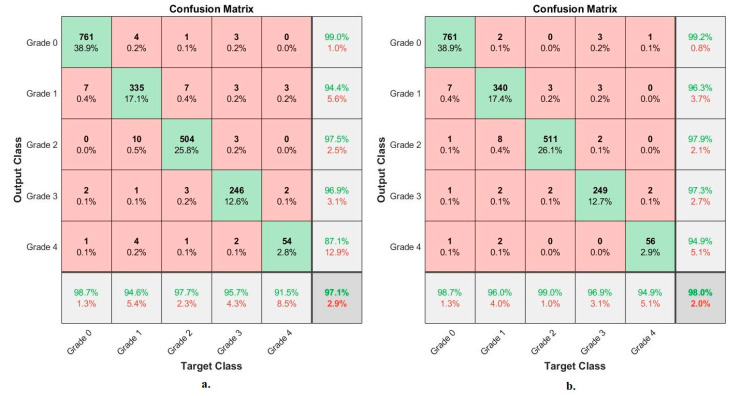
Display of the confusion matrix of the X-ray analysis of an OAI dataset for early diagnosis of severe KOA by FFNN with fusion features of (**a**) VGG-19-ResNet-101-PCA (**b**) VGG-19-PCA with ResNet-101-PCA.

Figure 10 shows the performance of FFNN based on the combined features of VGG-19 and ResNet-101 of the OAI dataset and severity grade discrimination. With hybrid features of VGG-19-ResNet-101-PCA, FFNN achieved the following accuracies for each grade to distinguish the severity of osteoarthritis: accuracy for Grade 0 of 98.7%, for Grade 1 of 94.6%, for Grade 2 of 97.7%, for Grade 3 of 95.7%, and for Grade 4 of 91.5%. Whereas, with hybrid features of VGG-19-PCA with ResNet-101-PCA, FFNN achieved the following accuracies for each grade to distinguish the severity of osteoarthritis: for Grade 0 of 98.7, for Grade 1 of 96%, for Grade 2 of 99%, for Grade 3 of 98.9%, and for Grade 4 of 94.9%.

**Figure 11:**
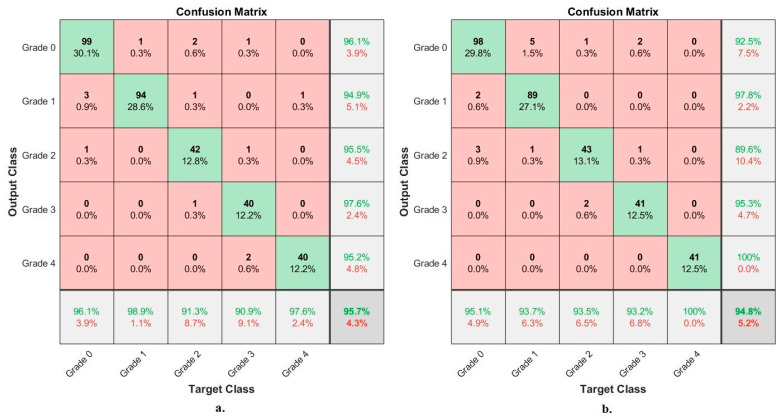
Display of the confusion matrix of the X-ray analysis of an RCU dataset for early diagnosis of severe KOA by FFNN with fusion features of (**a**) VGG-19-ResNet-101-PCA (**b**) VGG-19-PCA with ResNet-101-PCA.

Figure 11 shows the performance of FFNN based on the combined features of VGG-19 and ResNet-101 of the RCU dataset and severity grade discrimination. With hybrid features of VGG-19-ResNet-101-PCA, FFNN achieved the following accuracies for each grade to distinguish the severity of osteoarthritis: accuracy for Grade 0 of 96.1%, for Grade 1 of 98.9%, for Grade 2 of 91.3%, for Grade 3 of 90.9, and for Grade 4 of 97.6%. Whereas, with hybrid features of VGG-19-PCA with ResNet-101-PCA, FFNN achieved the following accuracies for each grade to distinguish the severity of osteoarthritis: for Grade 0 of 95.1%, for Grade 1 of 93.7%, for Grade 2 of 93.5%, for Grade 3 of 93.2%, and for Grade 4 of 100%.

### 4.6. Results of FFNN with Hybrid Features of CNN and Handcrafted Features

This section summarizes the results of hybrid systems with fusion features for X-ray image analysis of OAI and RCU datasets to diagnose the severity of osteoarthritis before it progresses to the severe stage. Two methods were developed by combining CNN features (VGG-19 and ResNet-101) separately with handcrafted features. This technique aims to extract the features of VGG-19 and ResNet-101 separately and then reduce the high dimensionality by PCA. The important features are sent to FFNN to split the features of the two datasets to train the systems and test their performance.

Table 8 summarizes the results obtained through FFNN based on the fusion features of the OAI dataset and severity grade discrimination. With the fusion features of the VGG-19-PCA and handcrafted features, FFNN reached an AUC of 99.25%, an accuracy of 99.1%, a sensitivity of 98.81%, a specificity of 100%, and precision of 98.24%. Whereas, with the fusion features of ResNet-101-PCA and handcrafted features, FFNN reached an AUC of 99.28%, an accuracy of 99%, a sensitivity of 97.96%, a specificity of 100%, and precision of 98.66%.

Table 9 summarizes the results obtained through FFNN based on the fusion features of the RCU dataset and severity grade discrimination. With the fusion features of the VGG-19-PCA and handcrafted features, FFNN reached an AUC of 99.07%, an accuracy of 98.20%, a sensitivity of 98.16%, a specificity of 99.73%, and precision of 98.08%. Whereas, with the fusion features of ResNet-101-PCA and handcrafted features, FFNN reached an AUC of 97.98%, an accuracy of 96.4%, a sensitivity of 95.90%, a specificity of 98.92%, and precision of 96.4%.

**Figure 12:**
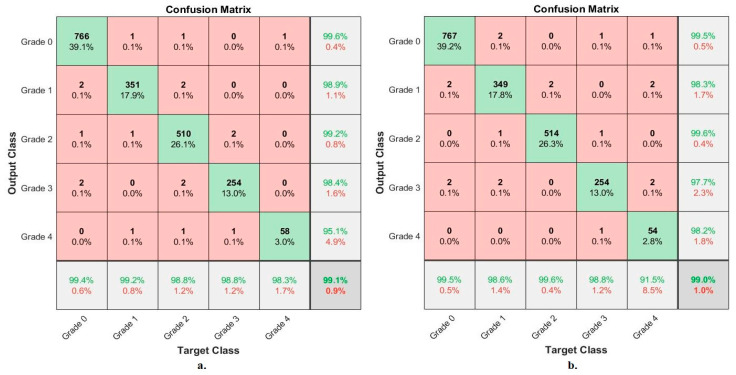
Display of the confusion matrix of the X-ray analysis of an OAI dataset for early diagnosis of severe KOA by FFNN with features of (**a**) VGG-19-PCA-handcrafted (**b**) ResNet-101-PCA-handcrafted.

Figure 12 shows the performance of FFNN based on the fusion features of the OAI dataset of osteoarthritis and severity grade discrimination. With fusion features of VGG-19-PCA and handcrafted features, FFNN achieved the following accuracies for each grade to distinguish the severity of osteoarthritis: accuracy for Grade 0 of 99.4%, for Grade 1 of 99.2%, for Grade 2 of 98.8%, for Grade 3 of 98.8%, and for Grade 4 of 98.3%. Whereas, with fusion features of ResNet-101-PCA and handcrafted features, FFNN achieved the following accuracies for each grade to distinguish the severity of osteoarthritis: for Grade 0 of 99.5, for Grade 1 of 98.6%, for Grade 2 of 99.6%, for Grade 3 of 98.8%, and for Grade 4 of 91.5%.

**Figure 13:**
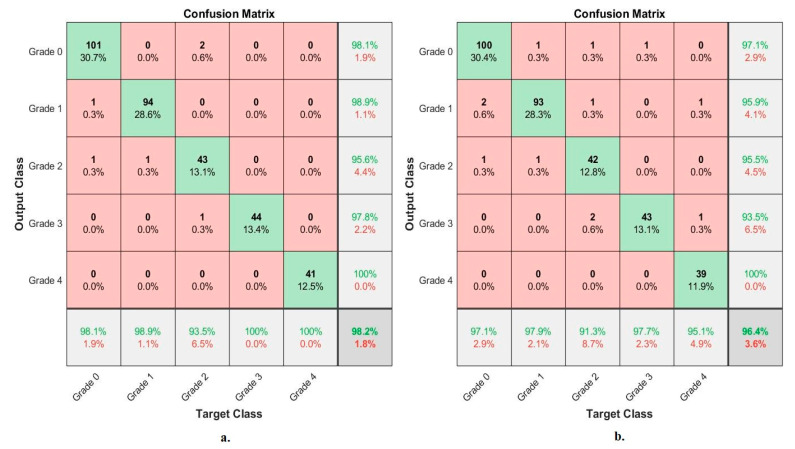
Display of the confusion matrix of the X-ray analysis of an RCU dataset for early diagnosis of severe KOA by FFNN with features of (**a**) VGG-19-PCA-handcrafted (**b**) ResNet-101-PCA-handcrafted.

Figure 13 shows the performance of FFNN based on the fusion features of the RCU dataset of osteoarthritis and severity grade discrimination. With fusion features of VGG-19-PCA and handcrafted features, FFNN achieved the following accuracies for each grade to distinguish the severity of osteoarthritis: accuracy for Grade 0 of 98.1%, for Grade 1 of 98.9%, for Grade 2 of 93.5%, for Grade 3 of 100%, and for Grade 4 of 100%. Whereas, with fusion features of ResNet-101-PCA and handcrafted features, FFNN achieved the following accuracies for each grade to distinguish the severity of osteoarthritis: for Grade 0 of 97.1, for Grade 1 of 97.9%, for Grade 2 of 91.3%, for Grade 3 of 97.7%, and for Grade 4 of 95.1%.

## 5. Discussion the Performance of the Systems and Comparison Results

Sheik et al. [35] RCNN-trained X-ray images of knee patients to diagnose the knee joint, reaching an accuracy of 98.51%. Simon et al. [36] trained the ResNet network through PyTorch to determine the severity of knee inflammation, which reached an AUC of 92%. Jiangling et al. [37] used an aggregated multiscale dilated convolutional network for feature learning, combined with aggregated multiscale dilated CNN, and achieved an accuracy of 93.6%. Dilovan et al. [38] presented a deep learning model to extract features from X-rays of KOA. These features are then sent to the SVM, Naive Bayes, and KNN machine learning classifiers. KNN with deep learning features achieved better results than the rest of the classifiers, which reached an accuracy of 90.01% and a specificity of 87.8%. Rabbia et al. [39] performed an extraction of features from the knee joint space by hybrid features using directed gradient graph and classification by Random Fores, and achieved an accuracy of 97%. Ashish et al. [40] performed a classification of knee inflammation severity images based on adjusting the force parameters and classifying them by Decision Tree, achieving an accuracy of 91%.

Here, we review the results of the systems and compare the performance as follows.

Knee osteoarthritis is one of the most common diseases of the musculoskeletal system that disturbs life, and it is a chronic disease that leads to disability, especially for the elderly [41]. This disease causes joint pain and knee weakness, and late diagnosis leads to joint replacement, which is very expensive [42]. KOA goes through many stages from grade 0 to grade 4, called KL-grading [43]. The initial stages of KL grading are similar. Therefore, manual diagnosis by doctors and experts cannot notice the exact symptoms and characteristics that distinguish each grade from the other [44]. Thus, deep learning techniques can extract subtle and hidden features that are not noticed by manual diagnosis [45]. In this study, three methodologies were developed; each methodology has two different systems for analyzing X-rays for KL-grading of KOA.

The X-rays of the OAI and RCU datasets contain noise and low contrast of the ROI. Thus, all X-rays were optimized to obtain accuracy in the following stages of medical image processing. Data augmentation was applied to increase the images of the two datasets to overcome the overfitting problems facing CNN and the dataset imbalance problem. In all methodologies, the OAI and RCU datasets were divided into 80% for the training and validation phases of the systems, and 20% was allocated for testing the performance of the systems.

In the first methodology, the improved X-rays were inputted into VGG-19 and ResNet101 to extract the subtle and hidden features separately. The PCA method receives the features for further improvement, eliminating the unimportant and redundant features and keeping the essential features. Features of VGG-19-PCA and ResNet-101-PCA are fed separately to FFNN for diagnosis. For the OAI dataset with the significant feature of VGG-19-PCA, FFNN achieved an accuracy of 95.8%, while with the essential feature of ResNet-101-PCA, it attained an accuracy of 95.1%. For the RCU dataset with the essential feature of VGG-19-PCA, FFNN attained an accuracy of 93.3%, while with the essential features of ResNet-101-PCA, it achieved an accuracy of 91.5%.

In the second methodology, the improved X-rays of the OAI and RCU datasets were inputted into the VGG-19 and ResNet-101 to extract the subtle and hidden features separately. For the first system of the second methodology, the features of VGG-19 and ResNet101 are merged and sent to PCA for further improvement. FFNN receives VGG-19-ResNet-101-PCA features for high-accuracy diagnostics. For the OAI dataset, FFNN attained an accuracy of 97.7%, while with the RCU dataset, FFNN attained an accuracy of 95.7%.

For the second system of the second methodology, the VGG-19 features are sent to PCA to delete the unimportant and redundant features and keep the essential features. Similarly, ResNet-101 features are sent to PCA to delete unimportant and redundant features and keep essential features. Then, the essential features are combined, called features of VGG-19-PCA with ResNet-101-PCA, and sent to FFNN for high-accuracy diagnosis. For the OAI dataset, FFNN attained an accuracy of 98%, while with the RCU dataset, FFNN attained an accuracy of 94.8%.

In the third methodology, the improved X-rays of the OAI and RCU datasets are entered into VGG-19 and ResNet-101 to extract subtle and hidden features separately. Handcrafted features from the GLCM, DWT, and LPB methods are extracted and combined. For the first system of the third methodology, the VGG-19 features are sent to the PCA to delete the unimportant and redundant features and keep the essential features and then combine them with the handcrafted features. This is called the fusion features. FFNN receives the fusion features to diagnose them with high accuracy. For the OAI dataset, FFNN achieved an accuracy of 99.1%, while with the RCU dataset, FFNN achieved an accuracy of 98.2%.

For the second system of the third methodology, the ResNet-101 features are sent to PCA to delete the unimportant and redundant features, keep the essential features, and then combine them with the handcrafted features. This is called the fusion features. FFNN receives the radiological features to diagnose them with high accuracy. For the OAI dataset, FFNN achieved an accuracy of 99%, while with the RCU dataset, FFNN achieved an accuracy of 96.4%.

Table 10 summarizes the results achieved by the proposed systems for X-ray analysis of the OAI and RCU datasets of osteoarthritis. The table summarizes the results of the systems and the accuracy of diagnosing each system for each grade in the OAI and RCU data sets. First, for the OAI dataset, the best accuracy for the grade 0 and grade 2 classes of 99.5% and 99.6%, respectively, was by FFNN with fusion features of ResNet-101 and handcrafted. The best accuracy for grade 1 and grade 4 classes of 99.2% and 98.3%, respectively, was by FFNN with fusion features of VGG-19 and handcrafted. The best accuracy for the grade 3 class of 98.8% was by FFNN with fusion features of VGG-19-handcrafted and ResNet-101-handcrafted.

Secondly, for the RCU dataset, the best accuracy for grade 0 of 98.1% was by FFNN with fusion features of VGG-19 and handcrafted, equally by FFNN with essential features of VGG-19. The best accuracy for grade 1 of 98.9% was by FFNN with fusion features of VGG-19 and handcrafted, equally by FFNN with hybrid features of VGG-19 and ResNet-101. The best accuracy for grade 2 of 93.5% was by FFNN with fusion features of VGG-19 and handcrafted, equally by FFNN with hybrid features of VGG-19 and ResNet-101. The best accuracy for grade 3 of 100% was by FFNN with fusion features of VGG-19 and handcrafted. The best accuracy for grade 4 of 100% was by FFNN with fusion features of VGG-19 and handcrafted, equally by FFNN with hybrid features of VGG-19 and ResNet-101.

It is noted that the results of the proposed systems are significantly superior to previous related studies.

It is noted that the results of the proposed systems are significantly superior to previous studies related to all measures of accuracy, sensitivity, specificity, and AUC.

## 6. Conclusions

Osteoarthritis of the knee is a chronic disease that impedes movement, especially in the elderly. Therefore, early diagnosis of knee injury is necessary to avoid its development to the advanced stages, which require the replacement of knee joints. This study developed three X-ray methodologies for analyzing two OAI and RCU datasets for diagnosing osteoarthritis and discriminating between KL grades. The first methodology for diagnosing the degree of osteoarthritis uses two-hybrid systems: VGG19-PCA-FFNN and ResNet101-PCA-FFNN. The second methodology for diagnosing the degree of osteoarthritis by FFNN is based on hybrid features of VGG-19 and ResNet-101 before and after PCA. The third methodology for diagnosing the degree of osteoarthritis by FFNN is based on the fusion features of CNN (VGG-19 and ResNet-101) and handcrafted features.

We conclude that the performance of FFNN with hybrid features between the handcrafted CNN models was better than its performance with only CNN features or with combined CNN features.

For the OAI dataset with fusion features of VGG-19 and handcrafted, FFNN reached an AUC of 99.25%, an accuracy of 99.1%, a sensitivity of 98.81%, a specificity of 100%, and a precision of 98.24%. For the RCU dataset with the fusion features of VGG-19 and handcrafted, the FFNN reached an AUC of 99.07%, an accuracy of 98.20%, a sensitivity of 98.16%, a specificity of 99.73%, and a precision of 98.08%.

## Figures and Tables

**Table 1:** Distribution and description of the two X-rays of the OAI and RCU datasets according to KL grading.

Types	Description of KL Grading	Data Sets	
		OAI	RCU
Grade 0	Healthy X-ray	3857	514
Grade 1	X-rays of doubtful narrowing of the joint with osteophytes tip over	1770	477
Grade 2	X-rays have minimal osteoarthritis containing joint space narrow with osteophytes	2578	232
Grade 3	X-rays have moderate osteoarthritis containing joint space stenosis, multiple osteophytes, and mild sclerosis	1286	221
Grade 4	X-rays have severe injuries containing large osteophytes and severe sclerosis with clear narrowing of the joints	295	206
Total		9786	1650

**Table 2:** Splitting the OAI and RCU datasets in all phases.

Datasets	OAI			RCU		
Phase	80% (80:20)		Testing 20%	80% (80:20)		Testing 20%
Classes	Training (80%)	Validation (20%)		Training (80%)	Validation (20%)	
Grade 0	2469	617	771	329	82	103
Grade 1	1133	283	354	306	76	95
Grade 2	1650	412	516	149	37	46
Grade 3	823	206	257	140	35	46
Grade 4	189	47	59	132	33	41

**Table 3:** A method for balancing and augmenting the X-ray data for the two OAI and RCU datasets of osteoarthritis.

Datasets	OAI					RCU				
Phase	Training Dataset					Training Dataset				
Classes	Grade 0	Grade 1	Grade 2	Grade 3	Grade 4	Grade 0	Grade 1	Grade 2	Grade 3	Grade 4
Bef-augm	2469	1133	1650	823	189	329	306	149	140	132
Aft-augm	4938	4532	4950	4938	4914	3290	3060	3278	3220	3168

**Table 4:** Results of implementing FFNN with VGG-19 and ResNet-101 features for X-ray analysis of an OAI dataset of osteoarthritis.

Techniques	Type of Class	AUC %	Accuracy %	Sensitivity %	Specificity %	Precision %
FFNN with features of VGG-19	Grade 0	96.32	98.2	98.12	98.47	97.6
	Grade 1	97.17	91.8	92.37	97.54	92.3
	Grade 2	96.86	96.7	97.45	99.11	98
	Grade 3	96.95	96.1	96.26	99.31	95.4
	Grade 4	97.1	79.7	80.75	99.28	77
	Average ratio	96.88	95.80	92.99	98.74	92.06
FFNN with features of ResNet-101	Grade 0	98.54	97.70	97.84	99.27	98.00
	Grade 1	98.12	89.80	90.41	98.30	90.30
	Grade 2	97.83	97.30	97.24	99.24	96.40
	Grade 3	96.84	94.20	95.94	98.78	95.70
	Grade 4	97.46	79.70	80.10	98.81	74.60
	Average ratio	97.76	95.10	92.31	98.88	91.00

**Table 5:** Results of implementing FFNN with VGG-19 and ResNet-101 features for X-ray analysis of an RCU dataset of osteoarthritis.

Techniques	Type of Class	AUC %	Accuracy %	Sensitivity %	Specificity %	Precision %
FFNN with features of VGG-19	Grade 0	97.52	98.1	98.37	96.58	93.5
	Grade 1	96.85	92.6	93.42	98.25	95.7
	Grade 2	95.68	89.1	89.4	95.79	80.4
	Grade 3	94.8	86.4	86.17	100	97.4
	Grade 4	97.61	95.1	94.82	100	100
	Average ratio	96.49	93.30	92.44	98.12	93.40
FFNN with features of ResNet-101	Grade 0	97.32	96.10	96.10	96.19	92.50
	Grade 1	95.16	89.50	89.24	97.89	94.40
	Grade 2	92.87	87.00	86.94	96.76	80.00
	Grade 3	94.72	88.60	89.20	99.12	92.90
	Grade 4	96.28	92.70	93.38	98.93	95.00
	Average ratio	95.27	91.50	90.97	97.78	90.96

**Table 6:** Results of implementing FFNN with fusion features of VGG-19 and ResNet-101 for X-ray analysis of an OAI dataset of osteoarthritis.

Techniques	Type of Class	AUC %	Accuracy %	Sensitivity %	Specificity %	Precision %
FFNN with features of VGG-19-ResNet-101-PCA	Grade 0	98.56	98.7	99.36	99.17	99
	Grade 1	96.48	94.6	95.43	98.88	94.4
	Grade 2	98.3	97.7	97.88	99.33	97.5
	Grade 3	97.83	95.7	96.11	100	96.9
	Grade 4	96.28	91.5	92.28	100	87.1
	Average ratio	97.49	97.10	96.21	99.48	94.98
FFNN with features of VGG-19-PCA with ResNet-101-PCA	Grade 0	98.91	98.70	99.14	99.31	99.20
	Grade 1	97.88	96.00	96.39	98.68	96.30
	Grade 2	97.56	99.00	98.87	99.29	97.90
	Grade 3	97.31	96.90	97.42	100	97.30
	Grade 4	96.62	94.90	94.76	100	94.90
	Average ratio	97.66	98.00	97.32	99.46	97.12

**Table 7:** Results of implementing FFNN with fusion features of VGG-19 and ResNet-101 for X-ray analysis of an RCU dataset of osteoarthritis.

Techniques	Type of Class	AUC %	Accuracy %	Sensitivity %	Specificity %	Precision %
FFNN with features of VGG-19-ResNet-101-PCA	Grade 0	97.1	96.10	96.13	97.88	96.10
	Grade 1	98.68	98.90	99.26	98.30	94.90
	Grade 2	94.26	91.30	91.42	99.11	95.50
	Grade 3	92.68	90.90	90.95	100	97.60
	Grade 4	98.74	97.60	97.89	99	95.20
	Average ratio	96.29	95.70	95.13	98.86	95.86
FFNN with features of VGG-19-PCA with ResNet-101-PCA	Grade 0	96.84	95.10	95.25	96.36	92.50
	Grade 1	96.22	93.70	94.37	99.41	97.80
	Grade 2	95.69	93.50	93.40	97.87	89.60
	Grade 3	97.81	93.20	92.96	99	95.30
	Grade 4	98.24	100	100	100	100
	Average ratio	96.96	95.70	95.20	98.55	95.04

**Table 8:** Results of implementing FFNN with fusion features of CNN-PCA-handcrafted for X-ray analysis of an OAI dataset of osteoarthritis.

Techniques	Type of Class	AUC %	Accuracy %	Sensitivity %	Specificity %	Precision %
FFNN with features of VGG-19 and handcrafted	Grade 0	99.3	99.4	99.25	100	99.6
	Grade 1	99.15	99.2	99.31	100	98.9
	Grade 2	99.81	98.8	98.74	100	99.2
	Grade 3	98.77	98.8	98.92	100	98.4
	Grade 4	99.24	98.3	97.82	100	95.1
	Average ratio	99.25	99.10	98.81	100.00	98.24
FFNN with features of ResNet-101 and handcrafted	Grade 0	99.46	99.50	99.40	100	99.50
	Grade 1	99.36	98.60	99.19	100	98.30
	Grade 2	99.64	99.60	100.00	100	99.60
	Grade 3	99.18	98.80	98.81	100	97.70
	Grade 4	98.76	91.50	92.42	100	98.20
	Average ratio	99.28	99.00	97.96	100	98.66

**Table 9:** Results of implementing FFNN with fusion features of CNN-PCA-handcrafted for X-ray analysis of an RCU dataset of osteoarthritis.

Techniques	Type of Class	AUC %	Accuracy %	Sensitivity %	Specificity %	Precision %
FFNN with features of VGG-19 and handcrafted	Grade 0	99.1	98.10	98.32	99.27	98.10
	Grade 1	98.85	98.90	99.11	100.00	98.90
	Grade 2	97.94	93.50	93.37	99.39	95.60
	Grade 3	99.45	100	100	100	97.80
	Grade 4	100	100	100	100	100
	Average ratio	99.07	98.20	98.16	99.73	98.08
FFNN with features of ResNet-101 and handcrafted	Grade 0	98.55	97.10	97.15	99.17	97.10
	Grade 1	97.87	97.90	98.36	97.59	95.90
	Grade 2	97.65	91.30	91.42	98.68	95.50
	Grade 3	98.11	97.70	97.84	99.18	93.50
	Grade 4	97.71	95.10	94.75	100	100
	Average ratio	97.98	96.40	95.90	98.92	96.40

**Table 10:** Summary of FFNN implementation performance of all systems for the X-ray analysis of the OAI and RCU datasets of osteoarthritis.

Datasets	Techniques		Features	Grade 0	Grade 1	Grade 2	Grade 3	Grade 4	Accuracy %
OAI Dataset	FFNN		VGG-19	98.2	91.8	96.7	96.1	79.7	95.8
			ResNet-101	97.7	89.8	97.3	94.2	79.7	95.1
	FFNN	Fusion features before PCA	VGG-19 with ResNet-101	98.7	94.6	97.7	95.7	91.5	97.1
		Fusion features after PCA	VGG-19 with ResNet-101	98.7	96	99	96.9	94.9	98
		Fusion features	VGG-19 and handcrafted	99.4	99.2	98.8	98.8	98.3	99.1
			ResNet-101 and handcrafted	99.5	98.6	99.6	98.8	91.5	99
**RCU Dataset**	FFNN		VGG-19	98.1	92.6	89.1	86.4	95.1	93.3
			ResNet-101	96.1	89.5	87	88.6	92.7	91.5
	FFNN	Fusion features before PCA	VGG-19 with ResNet-101	96.1	98.9	91.3	90.9	97.6	95.7
		Fusion features after PCA	VGG-19 with ResNet-101	95.1	93.7	93.5	93.2	100	94.8
		Fusion features	VGG-19 and handcrafted	98.1	98.9	93.5	100	100	98.2
			ResNet-101 and handcrafted	97.1	97.9	91.3	97.7	96.1	96.4

## Data Availability

X-ray images supporting the performance of the systems were obtained from two publicly available online datasets at the following links: -https://www.kaggle.com/datasets/tommyngx/kneeoa. -https://www.kaggle.com/datasets/tommyngx/digital-knee-xray?select=MedicalExpert-I (accessed on 15 December 2022).

## Abbreviations

CNN	Convolutional Neural Network
KOA	Knee Osteoarthritis
KL	Kellgren-Lawrence
OAI	Osteoporosis Initiative
PCA	Principal Component Analysis
FFNN	Feed Forward Neural Network
RCU	Rani Channamma University
CT	Computed Tomography
PET	Positron Emission Tomography
CLAHE	Contrast-limited Adaptive Histogram Equalization
DWT	Discrete Wavelet Transform
GLCM	gray-level Co-occurrence Matrix
LBP	Local Binary Patterns
AUC	Area under the ROC Curve
